# Governing AI in Electricity Systems: Reflections on the EU Artificial Intelligence Bill

**DOI:** 10.3389/frai.2021.690237

**Published:** 2021-07-30

**Authors:** Irene Niet, Rinie van Est, Frank Veraart

**Affiliations:** ^1^ Department of Industrial Engineering and Innovation Sciences, Eindhoven University of Technology, Eindhoven, Netherlands; ^2^ Rathenau Instituut, The Hague, Netherlands

**Keywords:** AI, electricity system, governance, autonomy, risks

## Abstract

The Proposal for an Artificial Intelligence Act, published by the European Commission in April 2021, marks a major step in the governance of artificial intelligence (AI). This paper examines the significance of this Act for the electricity sector, specifically investigating to what extent the current European Union Bill addresses the societal and governance challenges posed by the use of AI that affects the tasks of system operators. For this we identify various options for the use of AI by system operators, as well as associated risks. AI has the potential to facilitate grid management, flexibility asset management and electricity market activities. Associated risks include lack of transparency, decline of human autonomy, cybersecurity, market dominance, and price manipulation on the electricity market. We determine to what extent the current bill pays attention to these identified risks and how the European Union intends to govern these risks. The proposed AI Act addresses well the issue of transparency and clarifying responsibilities, but pays too little attention to risks related to human autonomy, cybersecurity, market dominance and price manipulation. We make some governance suggestions to address those gaps.

## Introduction

Based on a broad stakeholder consultation, the European Commission (EC) published the Proposal for a Regulation on a European approach for Artificial Intelligence (the “Artificial Intelligence Act”) in April 2021 (European Commission, 2018; European Commission, 2021). This bill addresses crucial aspects of governing artificial intelligence (AI). First, the proposal addresses the need and urgency of implanting regulations before AI systems are placed on the market “to ensure safety and respect of existing legislation protecting fundamental rights throughout the whole AI systems’ lifecycle” (European Commission, 2021). Second, the regulation gives a definition of AI^1^, which is necessary because there are debates about which digital technologies qualify as AI or not (Johnston, 2008; Poole and Mackworth, 2010; Sarangi and Sharma, 2019).

**FIGURE 1:**
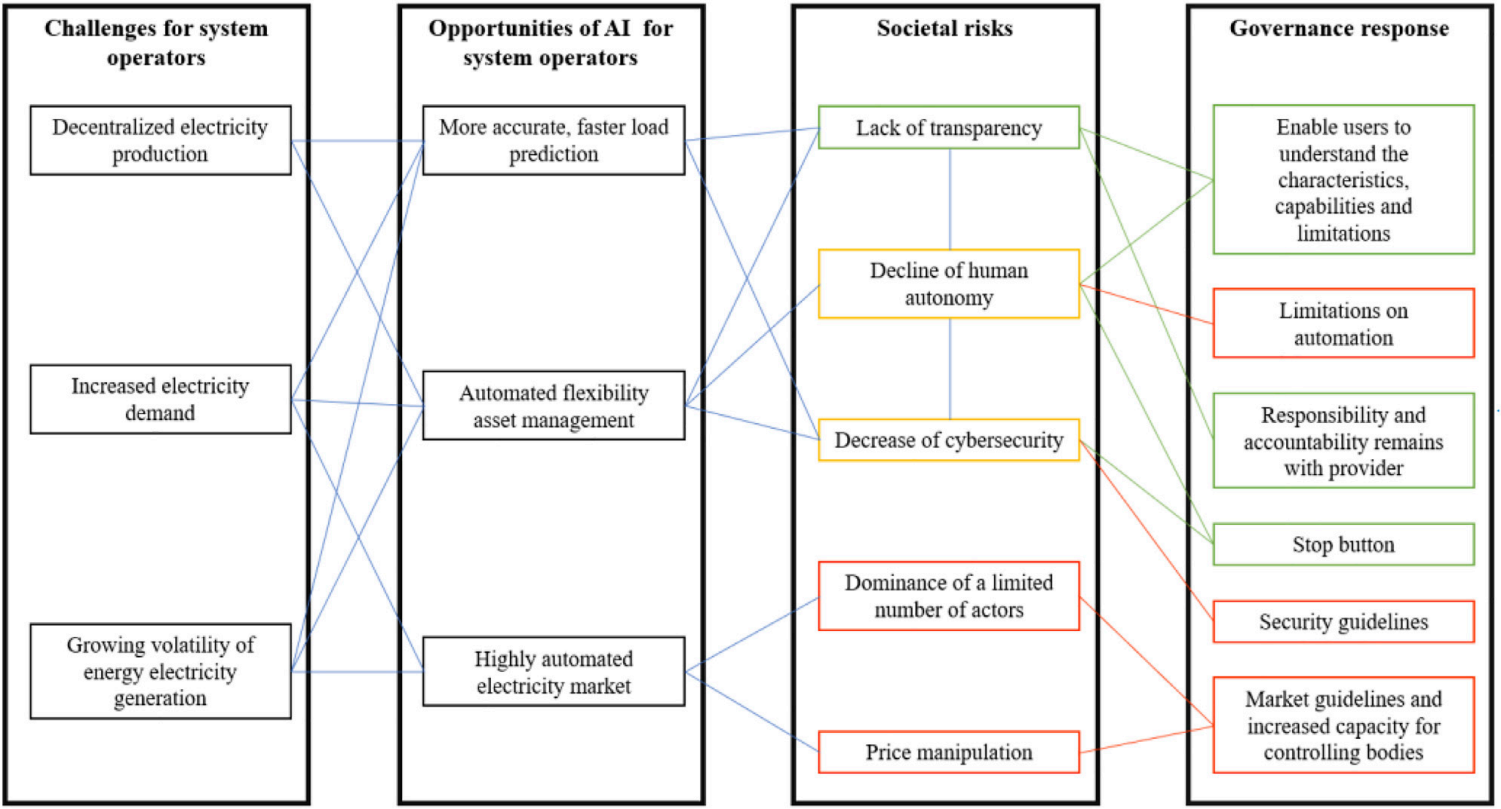
Overview of selected opportunities and risks of AI applications to support system operators, with related governance responses. Green risks are addressed in the EC’s Artificial Intelligence Act proposal. Yellow risks are partially addressed by the EC’s proposal. Red risks are unaddressed by the EC’s proposal. Green governance responses are responses proposed in the EC’s proposal. Red governance responses are governance response suggestions by the authors for those risks insufficiently addressed in the proposal. Source: authors.

The proposal for the AI Act presents a major step in the governance of AI. This paper examines the significance of this Act for the electricity sector, specifically by investigating to what extent the current European Union (EU) Bill pays attention to the societal and governance challenges posed by the use of AI affecting the tasks of System Operators (SOs). Below, we describe how the energy transition challenges SOs, how AI can be of use and what risks are associated with it. We then describe how the EU intends to manage these risks. Finally, we suggest ways to address identified gaps in the proposed legislation. In this way, this article aims to contribute to the discussion on governance of AI and the establishment of adequate European regulations in that area. Figure 1 offers an overview of the growing challenges for SOs, opportunities and risks of AI for SOs, how the proposal for the AI Act already handles some of these challenges and options for addressing underemphasized challenges.

## Challenges for System Operators

System operators are public utilities responsible for planning, building and maintaining the electricity distribution or transmission network, and providing a fair electricity market and network connections (MIT, 2016; Edens, 2017). Their goal is to keep the network reliable and secure, electricity affordable and making the system increasingly sustainable. Due to growing variable renewable energy production^2^ and the long-term expected increase in electricity demand^3^, SOs face new challenges with carrying out their public utility functions (see Figure 1, first column). The first challenge that SOs face is that, in contrast to the currently still dominant, more centralized ways of energy production, renewable energy production is decentralized, happening at various locations by a multitude of actors, using a variety of technologies (Dekker and van Est, 2020). For example, energy cooperatives are emerging that manage local, renewable energy projects (Delea and Casazza, 2010; Xu et al., 2019). Second, the production of electricity from renewable energy sources is volatile; their energy output is weather dependent (Bradford, 2018). Third, due to electrification, the demand for electricity is increasing and growing number of sectors rely on stable electricity supply^3^. The combination of volatile, decentralized electricity production and a growing electricity demand, has made it more complex for SOs to manage the power grid (MIT, 2016; van de Graaf and Sovacool, 2020).

## Opportunities of AI for System Operators

In response to these challenges, corporations from the energy sector, including SOs, and information technology sector are developing AI applications to support SOs in their utility function. Due to their ability to generate outputs such as content, predictions, recommendations, or decisions, AI offers many opportunities for SOs (see Figure 1, second column). To start, AI can be applied for more accurate load forecasting. The advantage of applying AI in the load forecasting system is two-fold. First, AI-based programs are able to include changes in the meteorological, social or economic context in their prediction models, resulting in more accurate short-term load forecasting (Zor et al., 2017; Al Mamun et al., 2020; Solyali, 2020), used by SOs for net balancing (Park et al., 1991; Kirby, 2005). Second, AI can improve long-term load forecasting, used by SOs to identify future bottlenecks and thus investment opportunities in the electricity grid (Park et al., 1991), by analyzing and “testing” the effectiveness of different investments before they are implemented, using digital twins (Onile et al., 2021).

The second opportunity of AI for SOs lies in simplified or even automated management of flexibility assets. Flexibility assets are technologies, such as home batteries and electric vehicles, with the ability to “save” electricity, providing flexibility for the electricity grid (Powells and Fell, 2019). SOs could make use of these technologies to support net balancing: when there is an oversupply of electricity on the grid, SOs could signal the flexibility assets to charge; with an undersupply, SOs could signal to discharge (Mbuwir et al., 2020). With their ability to give precise, local overviews of the flexibility capacity available, AI-based programs could support SOs’ manual flexibility management (Esmat et al., 2018; Radecke et al., 2019). Alternatively, flexibility management could be automated: AI could be applied to balance the electricity net autonomously without human involvement (Shen et al., 2018; Frendo et al., 2020). Small-scale experiments in which AI-based programs are taking over the tasks of an SO within a micro-grid are already taking place (Reijnders et al., 2020).

Third, AI can be applied to support or carry out electricity market activities, creating a highly automated electricity market. As described above, AI-based programs can estimate electricity prices on the basis of the prediction of electricity supply and demand (Xu et al., 2019; Qiao and Yang, 2020). Although this can be used to improve human decision-making on the electricity market, the great opportunity of AI lies in automated, near-real-time electricity trade. AI could predict fluctuations in the electricity market prices and manage its flexibility assets accordingly (Pinto et al., 2019). When electricity prices fluctuate (for example, rise due to an undersupply), AI-based programs can react (by discharging electricity from their flexibility assets, and selling this electricity for a higher price) and in doing so, re-balance the grid (Pinto et al., 2019; Xu et al., 2019). Electricity grid balancing based on electricity market price fluctuations is already taking place but currently works imperfectly, as oversupply of (renewably generated) electricity is curtailed instead of saved (Burke and O’Malley, 2011; Bird et al., 2016). Experiments with micro-grids have shown that AI-based programs are capable of autonomously managing flexibility assets to prevent oversupply on the electricity grid (Hou et al., 2019; Reijnders et al., 2020).

## Societal Risks and the European Commission’s Anticipation

There are various societal concerns with applying AI to the electricity system (see Figure 1, third column). Some of these highly probable risks with major societal impact are already addressed by the EC’s proposal for the AI Act (see Figure 1, column 4, green), but others have remained unaddressed (see Figure 1, column 4, red). The first concern regards the lack of transparency, which could lead to accountability issues. Although the electricity system has always been complex, the application of AI intensifies this (Delea and Casazza, 2010; van de Graaf and Sovacool, 2020). System operators frequently purchase AI technology (or service) from IT companies and startups (Makris et al., 2018; Mahmud et al., 2020; Mucha and Seppala, 2020). SOs use the program, but are often no experts in how the program operates; it is a black box. Such a situation is already happening in some micro-grids (Kloppenburg and Boekelo, 2019; Reijnders et al., 2020). This can result in SOs making decisions (regarding balancing or investments) based on models that they do not understand or control, leading to questions regarding accountability for public spending, high electricity prices or network downtime (MIT, 2016; Doran et al., 2017). For accountability purposes and to prevent automation bias or “overtrusting” the program (Kalayci et al., 2021), it should be clear on what basis data and data-analyses decisions are made.

In the proposal for the AI Act, the subject of transparency is discussed in great length. Article 13 of Chapter 2 of the proposal addresses the need for transparency, defining it as understanding the characteristics, capabilities and limitations of the AI program (European Commission, 2021). It is deemed the task of the legal person placing the AI on the market or putting it into service under its own name or trademark to ensure this understanding with the users of the AI program, and to guarantee enough human oversight for the system to minimize automation bias (European Commission, 2021, Article 13–14). The information for users should be “concise, complete, correct and clear” as well as “relevant, accessible and comprehensible” (European Commission, 2021, Article 13). This guideline covers most concerns, and should prevent incomprehensible and extensive terms-of-service agreements.

Second, the application of AI might limit human autonomy. Using AI for automated flexibility asset management instead of supporting SOs “manual” flexibility management leaves SOs with limited or no options regarding flexibility management, and obstructs SOs in differentiating from the pre-programmed path (Danaher, 2019; Lyytinen et al., 2020). Overriding of the program can be necessary in case of bias or cyberattacks. It can, however, be challenging for SOs to adjust the AI-based program in use, as it might not be owned or developed by them (Lin and Bergmann, 2016; Gunduz and Das, 2020).

The risk of limiting human autonomy is only partially addressed in the AI Bill. The proposal mentions that intervention in the AI program should always be possible and that AI subliminally distorting people’s behavior in a way that is likely to cause physical or psychological harm is prohibited (European Commission, 2021, Article 5, Article 14). Additionally, ensuring understanding with users of AI programs, such as SOs, supports human autonomy (Milchram et al., 2020). No guidelines are, however, included to limit automation, making decreasing human freedom to the set pre-programmed path of an AI still a possibility (Lyytinen et al., 2020).

The third risk concerns cybersecurity. The increase of renewable energy and electrification leads to more devices connected to the grid and, via their AI program, connected to the internet. AI programs require two-way communication: the program gathers data (such as electricity consumption) and sends commands (for example, a signal to an electric vehicle to charge) (MIT, 2016). These open networks are more vulnerable to non-authorized access or other types of disruption (such as false data injection) than one-way communication systems^4^ (Lin and Bergmann, 2016; Ryoo et al., 2017; Chehri et al., 2021; Zhuang et al., 2021). As AI-based programs can make autonomous decisions directly affecting the electricity grid, faulty decisions resulting from cyberattacks should be prevented, but a previously confirmed successful attack on the European Network of Transmission System Operators for Electricity has proven that this is not always possible (Khatiri-Doost and Amirahmadi, 2017; ENTSO-E, 2020; Yamin et al., 2021). Interestingly enough, by using AI for real-time monitoring of the electricity infrastructure it can also be used to increase cybersecurity (Mohammadpourfard et al., 2020). Developing a monitoring program that includes the growing number of cybersecurity threats is, however, difficult. The goal of such a monitoring program would be to exclude malicious access and use, but not exclude or slow down the various forms of legitimate access from the growing number of decentralized electricity generators, flexibility assets and aggregators (Balda et al., 2017; Philips et al., 2021).

The EC discusses cybersecurity in the AI Bill, but not in great detail. They mention that “[h]igh-risk AI systems should perform consistently throughout their lifecycle and meet an appropriate level of accuracy, robustness and cybersecurity in accordance with the generally acknowledged state of the art” (European Commission, 2021). Additionally, the system should be “resilient” against unauthorized access (European Commission, 2021, Article 15). There are, however, no parameters for these requirements. The requirement of a stop button for the AI-based program increases cybersecurity, but works after a security breach, instead of being a preventive measure (Taddeo et al., 2019). Apart from this, there are references to previous regulations, but these, too, lack specific guidelines for electricity systems (European Parliament and Council, 2019).

The last two risks relate to the functioning of the electricity market. The fourth risk is dominance of a limited number of actors due to platformization. AI-based energy platforms are emerging that offer electricity use and flexibility services (Kloppenburg and Boekelo, 2019). In some sectors, platformization has followed a winner-takes-all principle; for example, Google is the major search engine (Moore and Tambini, 2018). Platformization is receptive for this principle, because bigger platforms are often able to offer better services at lower prices compared to smaller platforms, as they can spread their costs over more users (van Dijck et al., 2016; Langley and Leyshon, 2017). Such dominance of a limited number of actors would distort the electricity market, as smaller energy platforms would be unable to compete (Kloppenburg and Boekelo, 2019). When this occurs creating a fair electricity market becomes more complex for SOs.

The fifth risk regards price manipulation on the electricity market. Due to the complexity of AI, it is often unknown on what basis AI programs operate, and the programs can be used by a variety of actors for different goals. SOs cannot monitor what data the AI uses to make decisions about the electricity market (Sarangi and Sharma, 2019). This could result in multiple AI programs conflicting with each other or with the goal of the SOs to create a reliable, affordable and sustainable electricity network^5^ (Petre et al., 2019). For example, AI programs supporting electricity buyers might be programmed to buy at the lowest price, whereas AI programs supporting electricity sellers might be programmed to sell at the highest price, leading to a delay in meeting electricity demand. Additionally, there is a risk that the buyer and seller become interlinked in one platform, resulting in the electricity seller prioritizing electricity buyers if they use the same platform service (Evans, 2012). An automated trading system could also emerge, with risks such as the growth of resellers which add no real value to the system, and flash markets in which demand and supply are highly volatile (Borch, 2016; Todorović et al., 2019). All of this could result in a highly unstable market with inflated prices, which can happen both intentionally and unintentionally (Karppi and Crawford, 2016).

In the proposal for the AI Act, the EC does not mention platformization or electricity market manipulation, although the legitimacy of the proposal and subsequent regulations is based on the legislation for the European Single Market (European Union, 2020). Electricity related platformization and market changes due to the integration of AI are also not mentioned in other recent proposals of the EC, such as the Digital Services Act (European Commission, 2020a) and the Digital Markets Act (European Commission, 2020b). This last proposal does mention “energy” as a sector with core platform services, with most evident and prominent problems in need of guidelines, but does not include such guidelines.

## Discussion: Underemphasized Risks and How to Mitigate These

From this analysis, we can conclude that with its proposal for the AI Act, the EC has already taken major steps in guiding the development and implementation of AI in critical or vital infrastructures, such as the electricity system. We discussed how AI can support SOs with the challenges of growing electricity demand and integration of renewable energy, but also introduce or contribute to various risks (see Figure 1). We analyzed how the proposal of the EC has addressed some of these risks. The challenges of lack of transparency and unclear division of responsibilities are well-addressed. Still, there are problems that have remained under-emphasized.

First, specific guidelines on limiting automation and increasing security regarding the use of AI in the electricity system are lacking. Such guidelines could offer SOs a way of enforcing (new) (cyber)security measures, ensuring human freedom and legitimate the safe use of AI-based programs (Gregory et al., 2020). For example, it is important that flexibility assets should adhere to certain security standards and protocols before being connected to the electricity grid. Additionally, even in fully automated systems, SOs should be able to differ from automated paths.

Regarding the risks of dominance of a limited number of actors due to platformization, and price manipulation, the EC could further develop electricity market guidelines. These guidelines could increase the capacity of controlling bodies, such as the EU Agency for the Cooperation of Energy Regulators and the Consumer Protection Cooperation Network. Together, these bodies could monitor the application of AI in the EU electricity market. However, these bodies need legal grounds for intervention, which are currently lacking.

Future research is necessary to clarify and keep up to date with emerging opportunities and risks for SOs of applying AI in the electricity system. The EU, national governments, regional institutions and SOs require an informed view to develop additional guidelines. Such improvements to the current proposal can aid to prevent or solve emerging public issues.

## Data Availability

The original contributions presented in the study are included in the article/Supplementary Material, further inquiries can be directed to the corresponding author.

## Footnotes

1 AI is defined as “software that is developed with [machine learning, logic- and knowledge-based, or statistical approaches], and can, for a given set of human-defined objectives, generate outputs such as content, predictions, recommendations, or decisions influencing the environments they interact with” (European Commission, 2021).

2 Based on the EU-27, the share of renewable energy in the gross final electricity production in the EU grew between 2000 and 2019 from 15.3 to 29.1% (Eurostat, 2021). The following Standard International Energy product classes are included as renewable: hydro, geothermal, wind, solar, and tide, wave, ocean. This gross production results in a net production of electricity and heat.

3 The IEA (2021) notes that “[g]lobal electricity demand increases by 80% between 2020 and 2050, around double the overall rate of growth in final energy consumption.” The authors acknowledge most of this increase comes from developing economies, but with the pledge for a net-zero economy, and the electrification of industry, buildings and transport, the electricity demand is also expected to rise in the EU (IEA, 2020; IEA, 2021).

4 There are many possibilities for cyber-attacks in electricity systems including digital technologies such as AI. For an overview, see Chehri et al. (2021) and Zhuang et al. (2021).

5 For a more detailed overview of how AI programs can affect markets, see Azzutti et al. (2021) and Fan et al. (2018).
